# General Considerations for On-Animal Ectoparasiticidal Product Evaluations

**DOI:** 10.1093/jisesa/ieaa042

**Published:** 2020-11-02

**Authors:** Brandon G Smythe, Ulises A Sanchez-Sandoval

**Affiliations:** Center for Animal Health and Food Safety, New Mexico State University, Las Cruces, NM

**Keywords:** ectoparasiticidal, study design, veterinary entomology

## Abstract

Successfully preparing for and conducting on-animal ectoparasiticidal evaluations is key in providing accurate results and inferences on product performance. However, the procedures associated with designing sound-reliable research projects while using animal test subjects can become complex. The current manuscript offers insights towards the characterization of different evaluation types highlighting key considerations and potential problematic barriers that may otherwise be overlooked by researchers new to the area of on-animal product evaluation. Furthermore, recommendations on reporting inferences from findings based on various study designs are discussed. The authors of the current manuscript offer these considerations in the hopes of maintaining harmony in future reports used to develop and evaluate on-animal ectoparsiticidal products in the field of veterinary entomology.

The complex parasitic interactions that drive large economic losses in agricultural animal production continue to provide a broad range of challenges for producers and researchers alike (Drummond et al. 1981, Hawkins 1993). In response to increased human population projections and associated animal food demands, efforts aimed at maximizing animal health, profitability, and performance will be key in meeting future societal needs (FAO 2009). Inherent to these efforts is the need to broaden current parasite managerial options, explore novel control programs, and effectively evaluate new and existing parasiticidal products in support of sustainable animal production in a market destined for expansion.

Parasiticidal intervention has long been an effective tool in combating the detrimental effects associated with a broad range of economically important animal pests (Pimentel 2009). Unfortunately, recurring issues with pest population resistance have restricted the effective performance of multiple marketed active ingredients (Bossard et al. 1998, Scott et al. 2013, Coles and Dryden 2014). In addition to maximizing the effectiveness of existing marketed products, it may be in the interest of researchers to explore nontraditional active ingredients for on-animal use (Zaim and Guillet 2002). In this case, product evaluation becomes important for screening, dose determination, and overall efficacies of candidate compounds and formulations. Regardless of individual objectives, on-animal product evaluations should be conducted in a safe, replicable, and reliable fashion.

The purpose of the current manuscript is to offer general guidelines that can be used to ensure consistent and reliable product efficacy determinations for both marketed and experimental products aimed at on-animal dosing and exposures. An all-inclusive effort which would transcend multiple pest species and even commodities is beyond the scope of the current effort. Moreover, available products, application types, and formulations across animal production systems are as vast as the species labeled for control. However, many of the concepts presented may be applicable on a case-by-case basis. For purposes of simplicity, the following guidelines are intentionally structured broadly around key approaches, concepts and considerations for future researchers attempting to undertake on-animal product testing. The authors in no way, challenge existing guidelines or recommendations. Rather, we offer insights within existing procedures that may be useful to novice researchers in the field of ectoparsiticide evaluations.

## Experimental Design

### Preliminary Steps

On-animal product assessments, as the name would imply, include the use of animal test subjects. Prior to any work being conducted on animal, appropriate regulatory (i.e., Institutional Use and Care Committee; IACUC) approval is necessary which includes the justification for use of live animals. Furthermore, the experimental use of agricultural animals generally falls under United States Department of Agriculture guidelines. Under no circumstances should animals be used for research purposes prior to or without the appropriate authoritative approvals. In fact, approvals are often required for publication in most scientific journals. There is a plethora of resources available to ensure animal welfare and established guidelines for certain species that are already in place (Holdsworth et al. 2006a, b; Marchiondo et al. 2007, 2013; Bobey 2015).

The first step to any on-animal product evaluation must begin by identifying specific research objectives. Many off-animal or animal alternative models might be more suitable for certain projects and should be considered. For instance, parasiticidal responses of given pest population can be assessed using laboratory based toxicological evaluations (Sheppard and Hinkle 1987, Crosby et al. 1991, Paramasivam and Selvi 2017). Such techniques bypass the unnecessary use of animal subjects while successfully addressing research interests and characterization of test populations. Regardless, when attempting to establish or demonstrate efficacies of new products or novel uses of existing products labeled for on-animal use, there are rarely any suitable alternative models available. The following section assumes researchers have considered alternative approaches and offers general considerations when planning for project initiation with regard to the characteristics of the animals, the products being used, testing location, and general labor requirements.

#### Animals

Animals will constitute experimental units driving statistical assessments of findings. As with all scientific investigations, the number of experimental units used to conduct a study should provide statistical power and reliability in support of the study design. Proper identification of the experimental units for any on-animal product testing is key. Typically, in field applications animals are treated individually but housed as a herd. In this case, the herd should serve as the experimental unit (Tempelman 2009). Therefore, multiple herds would be required to successfully acquire statistical replication. Herd evaluations within a single season would require multiple treatment and control herds for reliable inferences. Alternatively, yearly evaluations can be conducted to achieve appropriate statistical replication. Individual animal numbers within a treatment herd will be dependent upon availability and general capabilities of specific research locations. Guidelines and commercially available software to help determine appropriate sample size to obtain statistical significance are available (Lenth 2007).

Less commonly, researchers may be afforded the ability to utilize individual animals as the experimental unit. In this case, a combination of housing, treatment, infestation, and assessments are made at the animal level. Typically, while animal numbers can be decreased in this scenario, labor associated with conducting these types of projects are increased. Regardless of the role of the animals used in on-animal product evaluations, individual animal descriptions are suggested to establish clarity of the procedures used for the reader. Physiological state, age, sex, and breed should be detailed for any experiment using animals. When using individual animals as the experimental unit, the authors suggest allocating treatments based on pretreatment population assessments and/or body weights of the animals.

#### Products

In general, the authors have identified two types of assessments that are heavily relied upon when conducting on-animal product testing; 1) General confirmational studies which include marketed product evaluation and assessments on product performance and 2) experimental evaluations of off-label or non-marketed products. When evaluating existing marketed products through confirmational studies, label directions will dictate procedures and use of the products as results and inferences should be structured around applicable product directions. Researchers might want to use these confirmational studies to provide insight on regional variations of product performance which would offer more detailed insight towards future recommendations. Additionally, confirmational studies can be structured to evaluate various rotational programs, integrated pest management assessments, and/or offer early detection of the onset of pest population resistance to various parasiticidal products.

The authors classify any off-label product use as an experimental evaluation. When researchers are conducting experimental evaluations as described above, characteristics of the procedures employed will need to be scientifically just and approved by regulatory groups with jurisdiction over specific research groups. However, specific research objectives can be dramatically broadened as the application and subsequent findings with regard to performance are no longer bound by label claims. General on-animal product development employs the use of experimental evaluations to screen novel compounds and/or formulations and explore dosage rates leading to cost effective large scale development. For any experimental evaluation, appropriate animal disposition should be clarified and approved prior to initiation.

#### Testing Location

Another common factor to consider when designing on-animal product evaluations would be animal housing and testing location. Modifications of existing facility structures to enhance on-animal product evaluation capabilities will rarely, if ever, be justified in budgetary requests. As such, many researchers interested in assessing product performance must design product assessment studies within the confines of specific environmental capabilities and limitations. Oftentimes, in the case of confirmational studies, collaborations with local animal producers become beneficial. In this case, researchers should ensure that the facility chosen for an on-animal product assessment provides the necessary working environment that would facilitate both animal and worker safety. In many cases, producer collaborations extend research potentials in areas where on-animal product evaluations would otherwise not be possible. The authors implore researchers working in this capacity to nurture these relationships by exhibiting professionalism in their interactions as they not only represent themselves, and their institution, but the entire field of researchers utilizing similar resources. Collaborative producers will continue to be valuable contributors to on-animal product evaluations and will most likely be the first to adopt novel approaches for wide-scale implementation.

#### Labor

A final key consideration when designing a study evaluating on-animal product performance is labor. Effective, safe, and appropriate animal husbandry during trials can become rather laborious. Under no circumstances, should an experiment be conducted using animals that will not be provided the most suitable methods to alleviate any undue pain or suffering. Ensuring animal and researcher safety often will require multiple individuals to be listed as study participants. Research leaders should properly plan for experienced personnel to be available or in attendance during each animal handling event.

Following the identification of key objectives and establishment of an appropriate study design, the authors suggest the development of a calendar of events structured around treatment application and other key data collection and animal handling events. This document should detail each activity and provide insight to potential scheduling conflicts with key personnel that could jeopardize successful completion of said activities or more importantly, compromise animal health.

### Procedures

A properly prepared on-animal product assessment protocol will ensure a successfully conducted research project. For purposes of the current document, no standard protocols or procedures are presented. Rather, an arguably over simplification of commonalities across a wide application of scenarios is presented.

### Pre-test Population Establishment and Assessment

Pretreatment population assessments are key in determining population reductions in response to product application or performance. When making claims or inferences on a products ability to effectively reduce an existing population associated with an animal(s), pretreatment population assessments are necessary. Guidelines for estimating population densities vary from pest to pest and techniques utilized by the researchers evaluating product performance should be validated or commonly accepted for the given pest of interest. Pretreatment population assessments should be conducted at a time prior to treatment application. It may be common and in the best interest of the researcher to conduct such an estimate on the same day as treatment application but prior to administration. If researchers are estimating populations from a subset of animals within a herd, effort should be made to sample from the same animals throughout the trial to minimize inter-animal variation in population densities.

### Posttreatment Population Assessments

The procedures used for posttreatment population assessments should mimic those used to establish pretreatment populations. Sampling times will be dictated by individual pest species biology and general constructs of the study. Additional data, such as animal performance, behavior or health can be incorporated in protocols in an attempt to demonstrate the positive effects as a result of effective pest population reduction.

### Documentation

Documentation of key events such as, treatment times, application rates, and population assessments is critical when justifying claims of performance for all types of on-animal product testing. This becomes particularly important when experimental evaluations are aimed at product registration or general label inclusions. If researchers are working in this capacity, strict adherence to Good Laboratory Practice (GLP; WHO 2009) or Good Clinical Practice (GCP; WHO 2005) guidelines should be pursued. Projects submitted to various regulatory agencies are open to investigations of findings and claims of product performance. The authors encourage researchers who are working under GCP and GLP guidelines to develop a clear understanding of these procedures and associated implications.

### Data Analysis

For simplicity sake, the authors will address two common metrics of determining effective product performance. The first, population reduction, is in regards to an animal(s) posttreatment pest population assessments as a percentage of the pretreatment population and is commonly used when evaluating repellent compounds (Mullens et al. 2017). Inferences on population reductions are independent of control numbers and offer researchers the ability to assess product performance without the use of a control herd. This may be important with trials involving pests that pose an immediate or extreme danger to the host animal inherently not allowing an ethical use of a control herd. In some cases, predetermined pest levels may dictate the timing of ethical intervention thereby eliminating the possibility of sustaining a control comparison. Regardless, inferences on reductions without the use of a control herd should be made with caution. Should posttreatment populations fail to return to pretreatment levels, population reduction strictly due to product performance may be questionable. Researchers should take great care in all inferences of population reductions.

Alternatively, general efficacy is another common metric of product performance. In this case, the use of and access to a control animal(s) is essential as efficacy calculations require relatable control population estimations (Abbott 1925). Inferences on product performance including duration of efficacy, dose determination, and product to product comparisons can be made with the use of a control comparison.

Control populations should also be scrutinized by researchers. Efficacy calculations can be misleading in cases where control performance is less than optimal and fail to accurately represent naturally occurring population levels. As such, statistical comparisons may be used in addition to efficacy calculations to highlight control performance. Application of appropriate statistical comparisons may highlight variation between samples that is overlooked when using efficacy calculations alone. Specific statistical models should be structured to account for study design. Statistical comparisons may help solidify efficacies but must also provide biological soundness.

It should be noted that researchers may become limited in their ability to design statistically sound approaches to evaluating efficacy in response to limited animal availability. Modifications of ideal study designs including animals serving as their own control and adjusting treatment schedules across time may be implemented to maximize available animals for testing purposes.

### Common Problems

#### Environmental Influences

A variety of environmental influences may impact findings and inferences regarding on-animal product performance (Raveton et al. 2006, Schmahl et al. 2009). Researchers should make efforts to document environmental characteristics of study locations throughout the life of the project. Ambient temperatures, relative humidity, and rainfall may impact product performance and should be documented. Claims between and within product performance without detailed environmental characteristics should be made with caution.

#### Adverse Events

When conducting on-animal product evaluations, researchers should incorporate scheduled health evaluations in an attempt to maximize animal welfare. If adverse events occur, effort should be made to determine whether or not the event was due to the test product.

#### Costs

On-animal product evaluations can become costly for a number of reasons. The following discussion draws from the authors own experiences conducting on-animal experimental evaluations. Dependent upon commodity, budgetary expenses associated with animal purchases used in experimental evaluations may account for as much as 60% of allocated budgets. The remainder of budgetary allocations are devoted to supplies (~10%) and labor (~30%). Furthermore, off-animal models of product performance (i.e., bioassay screenings) can be conducted for less than 1/10th of those conducted directly on-animal (personal experience, BGS). Budgetary expenses will undoubtedly vary between researchers and locations. However, when preparing to conduct an on-animal product evaluation, researchers should ensure that the cost associated with safely and accurately conducting the study is reflective in appropriate funding requests.

## Results

The results of any on-animal product assessments can take many different shapes depending upon variables of interest and related research objectives. Therefore, comprehensive recommendations for providing research findings is beyond the scope of the current effort. Rather, general guidelines for considerations are provided below to in an attempt to capture a broad range of measurements taken.

Pest population descriptions are key in product assessments and require special attention when inferring product performance based on findings. Pretreatment pest populations should always be reported to detail natural occurring densities. Such assessments are particularly important when attempting to demonstrate population reductions in response to product application without the use of a control. In cases such as these, researchers may report reductions but must always disclose the reasons for control exclusion. Furthermore, inferences regarding the duration of reductions or product to product comparisons using observed reductions must be done with great caution.

The use of efficacy calculations and subsequent inferences on product performance in terms of pest population management should be restricted to research studies which utilize controls. Inferences between and within labeled on-animal products should additionally be presented in relation to labeled claims (i.e., how well a product worked versus claims of product), pretreatment population reductions, and duration of efficacies based on suitable control performance can be reported.

## Discussion

There are a number of reasons for researchers to pursue on-animal product evaluations which make providing specific guidelines difficult. In the present manuscript we provide generalized concepts, not in an attempt to displace existing guidelines established for individual pests or commodities, but to offer additional considerations for new researchers pursuing objectives related to on-animal product evaluations. The utilization of on-animal investigations to evaluate ectoparasiticidal compounds will continue to benefit long-term integrated pest management options for animal producers. Researchers in this area must take care to ensure that any animal use is justified and will provide contributions to further understanding the plethora of host-pest interactions that drive economic distress in animal production. It is our hope that the provided considerations along with existing recommendations will assist future researchers by offering these insights largely based on personal experiences.
